# Effects of Glucomannan Supplementation on Type II Diabetes Mellitus in Humans: A Meta-Analysis

**DOI:** 10.3390/nu15030601

**Published:** 2023-01-24

**Authors:** Zhanzhi Zhang, Yu Zhang, Xiaomei Tao, Yuying Wang, Benqiang Rao, Hanping Shi

**Affiliations:** 1 Department of General Surgery, Beijing Shijitan Hospital, Capital Medical University, Beijing 100038, China; 2 Key Laboratory of Cancer FSMP for State Market Regulation, Beijing 100038, China; 3 Department of VIP Medical Services, National Cancer Center, National Clinical Research Center for Cancer, Cancer Hospital, Chinese Academy of Medical Sciences and Peking Union Medical College, Beijing 100021, China; 4 Department of Pharmacy, Beijing Shijitan Hospital, Capital Medical University, Beijing 100038, China

**Keywords:** type II diabetes mellitus, glucomannan, nutritional intervention, meta-analysis, RCTs

## Abstract

The hypoglycemic and lipid-lowering effects of glucomannan are widely known, and it is a potential effective treatment for type II diabetes. In this study, we evaluated the effects of glucomannan supplementation on blood-lipid-related indicators, blood-glucose-related indicators, blood pressure (BP), and body weight (BW) in patients suffering from type II diabetes. We searched databases including PubMed, Cochrane, the comprehensive biomedical research database (Embase), Web of Science, and China National Knowledge Infrastructure (CNKI) for literature on glucomannan and type II diabetes. Six randomized controlled trials (RCTs) were eligible (*n* = 440 participants) to be included in our analysis. Glucomannan not only reduced the total cholesterol (TC) (MD −0.38 [95% CI: −0.61, −0.15], *p* = 0.001) and low-density lipoprotein (LDL) levels (MD −0.35 [95% CI: −0.52, −0.17], *p* < 0.0001) compared with the control group, but also reduced the fasting blood glucose (FBG) (MD −1.08 [95% CI: −1.65, −0.50], *p* = 0.0002), 2 h postprandial blood glucose (P2hBG) (MD −1.92 [95% CI: −3.19, −0.65], *p* = 0.003), fasting insulin (FINS) (MD −1.59 [95% CI: −2.69, −0.50], *p* = 0.004), and serum fructosamine (SFRA) levels (SMD −1.19 [95% CI: −1.74, −0.64], *p* < 0.0001). Our analysis indicates that glucomannan is an effective nutritional intervention for type II diabetes.

## 1. Introduction

**Figure 1:**
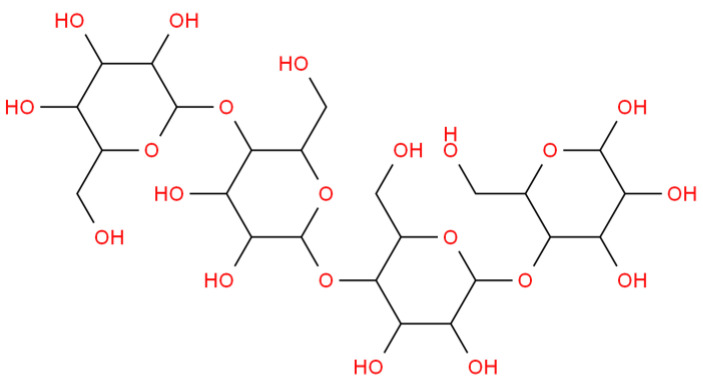
Chemical Structure of Glucomannan.

Glucomannan is a type of soluble hemicellulose. It has clear hypoglycemic and lipid-lowering effects, and its mechanism may be related to its rheological properties [1]. As a soluble dietary fiber, glucomannan becomes a viscous fiber with high viscosity after absorbing water, thus reducing the digestion and absorption of food, delaying the gastric emptying time, and reducing the postprandial blood glucose level [2]. Glucomannan is hydrophilic and contains a main chain polymerized by D-glucose and D-mannose through a β-l,4-glycosidic bond (Figure 1) and a branch chain polymerized by a β-1,3-glycosidic bond on the mannose of the main chain. Konjac, which is rich in fiber, contains a high amount of glucomannan. It is the main source of pure, natural, and high-molecular-weight glucomannan, which is a white powder. The refined powder processed from konjac generally contains more than 65% glucomannan and can reach approximately 80% using improved technology [3]. Glucomannan is a natural macromolecular compound with high water absorption and expansibility. It can form a viscous solution when dissolved in cold water, making it thick, emulsifiable, and suspended. When the pH value is below 12.2, it forms a reversible gel. When the pH is above 12.2 and heated, it forms an elastic gel, which is rare in other polysaccharides [4].

*Amorphophallus konjac* is a perennial herb of Amorphophallus Blume of Araceae [5]. Its bulb is the most used part. The water content in fresh bulbs is approximately 77%, and the water content is related to the plant cultivar, growth period, distribution area, cultivation management, and other factors. Polysaccharides account for approximately 70% of konjac dry matter, mainly glucomannan or starch [6]. Konjac is the only plant with a high content of glucomannan found so far worldwide, and the content of glucomannan in the bulb can reach about 50% of its dry weight [7]. Konjac glucomannan is another rich, natural, and renewable polysaccharide resource similar to starch and cellulose that has excellent biocompatibility and biodegradability [8].

Glucomannan is a high-quality dietary fiber and a safe food additive [9]. Glucomannan can aggregate with water molecules through hydrogen bonds, molecular dipoles, instantaneous dipoles, induced dipoles, and other forces to form large molecules that cannot move freely. In the dissolution process, the diffusion and migration speed of water molecules are far greater than those of glucomannan macromolecules, which makes glucomannan gum particles swell or expand to 80–100 times their original volume [10]. When glucomannan gel is heated under alkaline conditions, the acetyl group on its molecular chain is removed to form a stable gel. Even if it is heated repeatedly at 100 °C, its gel strength is unchanged [10]. This unique property is very rare in the field of polymer polysaccharides. In addition, glucomannan gel can still maintain an effective structure even after dialysis [11]. Therefore, utilizing this property in the food processing industry can effectively maintain the shape of a product without large deformation due to temperature changes. A study showed that the viscosity of glucomannan solution was much higher than that of carrageenan, xanthan gum, arabic gum, and other thickeners at the same concentration [11]. Unlike guar gum, xanthan gum, locust bean gum, and other thickeners, glucomannan is a nonionic thickener, which is relatively less affected by salt ions in the system, so it has important application value in the food industry.

The main difference between type Ι and type II diabetes is the presence of insulin resistance [12]. The International Diabetes Federation (IDF) predicts that by 2045, about 693 million adults will suffer from diabetes and its complications to various degrees [13]. Serious complications of diabetes, such as uremia caused by diabetic nephropathy, blindness caused by diabetic retinopathy, and the amputation of lower limbs due to diabetic foot, have a substantial impact on human health [14]. Diabetes requires extensive use of medical resources. Studies have shown that dietary fiber can alleviate type II diabetes and that high-viscosity dietary fiber further improves the treatment effect [15]. The glucomannan we studied is such a dietary fiber with high viscosity that is five times higher than that of guar gum and β-dextran [16]. Compared with its use for nearly 1000 years in East Asian countries, konjac glucomannan has only recently been used as dietary fiber in European and American countries, prompting food and health departments to study its safety and effectiveness [16]. In 2010, the European Food Safety Agency confirmed that konjac glucomannan is beneficial for weight loss, reducing postprandial blood sugar, and lowering blood cholesterol concentration, and issued a health statement [17]. In 2018, Kang found that konjac glucomannan remarkably reduced the BMI, fat mass, and serum triglyceride in obese patients. Many animal models confirmed glucomannan’s protective effect on diabetes and its complications [18,19]. However, the results in the relevant literature failed to reach a consensus. For example, konjac glucomannan significantly reduced fasting glucose in several studies, but Vuksan et al. showed that there was no significant difference. Currently there is no meta-analysis on the effects of glucomannan on type II diabetes based on randomized controlled trials (RCTs). Thus, we performed a meta-analysis of glucomannan supplementation on type II diabetes patients (experimental group) compared with controls or pre-treatment and found it could reduce TC, LDL-C, FBG, P2hBG, FINS, and SFRA in type II diabetic patients.

## 2. Materials and Methods

### 2.1. Search Strategy

The English and Chinese databases, including Embase, PubMed, Cochrane, Web of Science, and CNKI, were searched for relevant studies among the initial studies up to the date of 31 December 2021. We used the MeSH terms “Glucomannan” and “Diabetes Mellitus” in the retrieval process. The complete search combinations used in the search on Web of Science were “TS = (Glucomannan OR Konjac glucomannan OR Konjac mannan)” and “TS = (Diabetes Mellitus OR Diabetes OR Diabetic)”. All language and publication types were included in the search.

### 2.2. Eligibility Criteria

Two independent researchers closely investigated each word of the title and each sentence of the abstract. We traced and downloaded the full text of the RCTs that meet these search standards. Two researchers analyzed the experiments used for detailed analysis and data extraction, with differences resolved through mutual consultation or discussion with a third independent author. The inclusion criterion was RCTs of glucomannan in patients with type II diabetes, and the exclusion criteria were cell and animal experiments, patents, conference speeches, retrospective analyses, nonrandomized controlled studies, reviews, and meta-analyses.

### 2.3. Data Extraction and Outcomes

The extracted basic data are included in Table 1. The blood lipid levels, blood glucose index, and results of the physical examination are shown in the Section 3. Two independent authors completed all data extraction and input. The mean and standard deviation had not been recorded in some RCTs. We obtained the required data according to the conversion formula as stated in a previous study [17]. We assessed the risk of deviation based on PRISMA recommendations. Differences were resolved between them or with a third reviewer.

### 2.4. Statistical Analysis and Quality Assessment

Review Manager 5.3 software (Cochrane, UK) was used for the meta-analysis. The standardized mean difference (SMD) and 95% confidence interval were the basic analysis parameters of the continuous variables. We used a random effects model to determine the value when I^2^ exceeded 50%, which we used for the heterogeneity analysis, and conducted sensitivity analysis to analyze the robustness and reliability of the merge results. When the I^2^ value did not exceed 50%, we used the fixed effects model; *p* values less than 0.05 were considered statistically significant. We assessed the risk of publication bias using funnel plots derived from the Cochrane bias risk assessment tool. We evaluated the quality of the six RCTs using seven criteria, i.e., random sequence generation, allocation concealment, patient and personnel blindness, outcome evaluator blindness, incomplete outcome data, selective reporting, and other biases not covered above. Deviation risk included three types, namely, “low risk”, “high risk”, and “uncertain risk”. The evaluation was based on the Cochrane manual.

### 2.5. Risk of Publication Bias

Stata/MP 17.0 software (Stata, USA) was used to perform Egger’s test to assess publication bias; *p* < 0.05 indicated significant publication bias.

## 3. Results

### 3.1. Subsection

#### 3.1.1. Search Results

**Figure 2:**
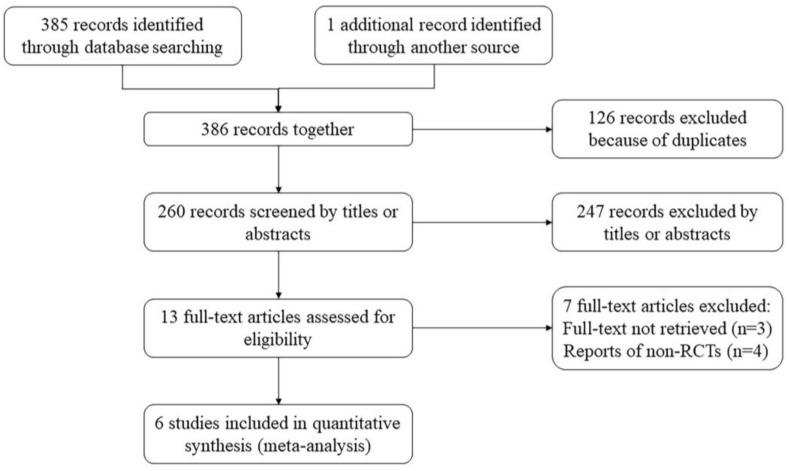
Flow chart of the study included in this systematic review.

We collected 198 studies from the Web of Science, 92 from Embase, 49 from PubMed, 25 from the Cochrane Library, 21 from CNKI, and 1 additional record from another source, with a total of 386 studies. After reviewing the titles and abstracts, we removed 126 duplicate articles and 247 unrelated articles, leaving 13 that met the requirements for searching their full texts. The full texts of three articles were not available to us. We read the remaining 10 articles and considered 4 of these to be RCTs in a unqualifying sense. Finally, there was a total of six RCTs of glucomannan on type II diabetes in the meta-analysis. The flow chart of the study is shown in Figure 2.

#### 3.1.2. Study Characteristics and Quality Assessment

**Figure 3:**
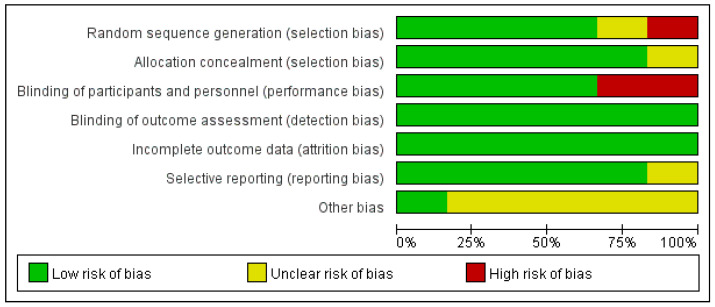
Quality Assessment of the Included Studies.

Among the six RCTs included in the meta-analysis, two were from China, two were from Canada, one was from Thailand, and one was from Taiwan [20,21,22,23,24,25]. All these data were published between 1999 and 2007. These included a total of 440 patients with type II diabetes. The number of people in the experimental group and the control group was the same (220 in each group). The results in Figure 3, obtained with the Cochrane bias risk tool, show the high quality of the six RCTs.

#### 3.1.3. The Effect of Glucomannan on Blood Lipids

1. Triglyceride (TG)

**Figure 4:**
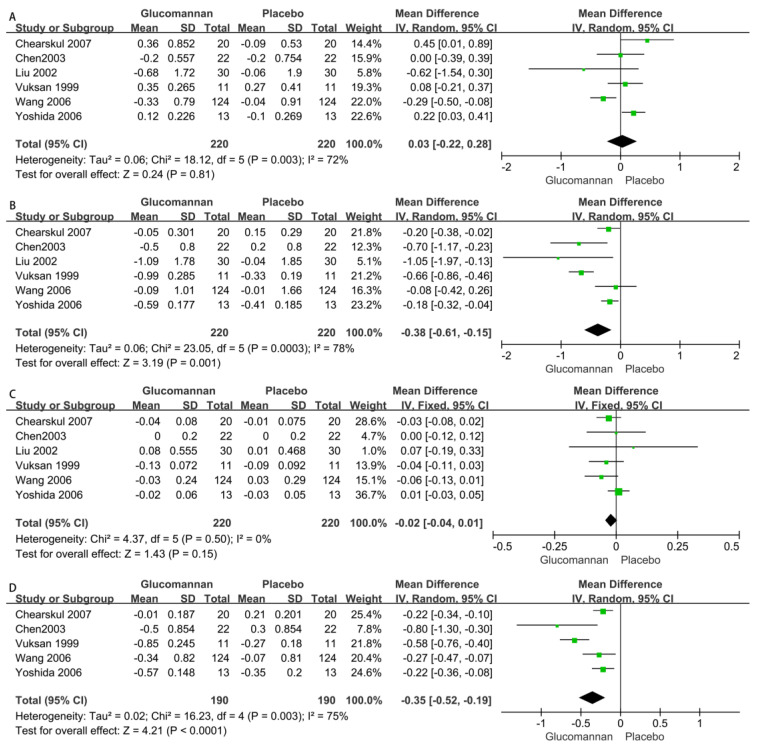
Forest plot of studies [20,21,22,23,24,25] on the effect of glucomannan intake on blood lipids included in this analysis. Effects of glucomannan on the TG (**A**), TC (**B**), HDL (**C**), and LDL (**D**) were analyzed, where green boxes indicate the odds ratio of the original research data and black diamonds indicate the overall odds ratio of studies included.

Six RCTs with a total of 440 patients, including 220 glucomannan users and 220 nonusers matching with the control group [20,21,22,23,24,25], showed that glucomannan did not significantly improve the TG levels (MD 0.03 [95% CI: −0.22, 0.28], *p* = 0.81). The heterogeneity among the included RCTs was significantly different (*p* = 0.003, I^2^ = 72%) (Figure 4A).

2. Total Cholesterol (TC)

Six RCTs with a total of 440 patients, i.e., 220 glucomannan users and 220 nonusers [20,21,22,23,24,25], showed that glucomannan reduced TC compared with controls, and the difference was statistically significant (MD −0.38 [95% CI: −0.61, −0.15], *p* = 0.001). The heterogeneity among the included RCTs was significantly different (*p* = 0.003, I^2^ = 78%) (Figure 4B).

3. High-Density Lipoprotein (HDL)

Six RCTs that reported the HDL levels of 440 patients, i.e., 220 glucomannan users and 220 nonusers [20,21,22,23,24,25], showed there was no significant difference between the two groups in the reduction in HDL levels (MD −0.02 [95% CI: −0.04, 0.01], *p* = 0.15) and the heterogeneity (*p* = 0.50, I^2^ = 0%) between the included RCTs (Figure 4C).

4. Low-Density Lipoprotein (LDL)

Five RCTs that reported the LDL levels of 380 patients, i.e., 190 glucomannan users and 190 nonusers [20,22,23,24,25], showed that compared with the control group, glucomannan reduced LDL levels, and the difference was significant (MD –0.35 [95% CI: −0.52, −0.19], *p* < 0.0001). The heterogeneity among the included RCTs was significantly different (*p* = 0.003, I^2^ = 75%) (Figure 4D).

#### 3.1.4. The Effect of Glucomannan on Glycemic Indices

1. Fasting Blood Glucose (FBG)

**Figure 5:**
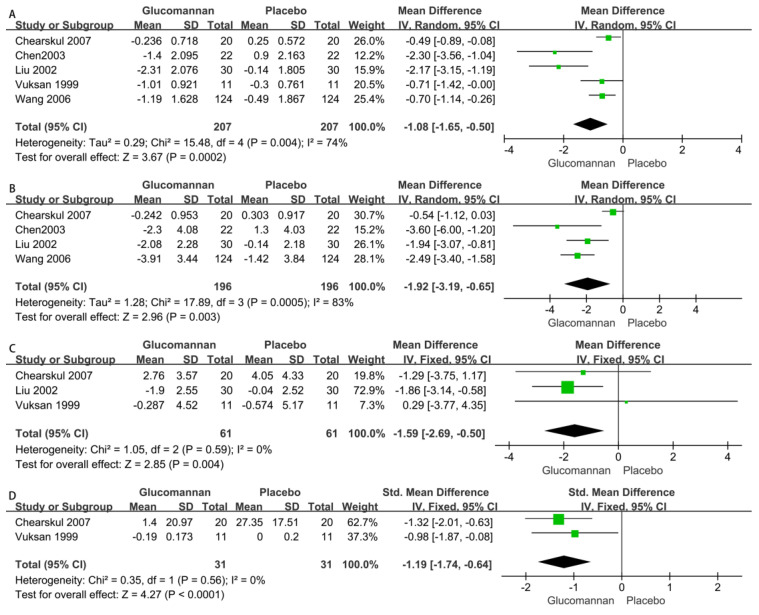
Forest plot of studies [20,21,22,23,25] on the effect of glucomannan intake on glycemic indices included in this analysis. Effects of glucomannan on FBG (**A**), P2hBG (**B**), FINS (**C**), and SFRA (**D**), where green boxes indicate the odds ratio of the original research data and black diamonds indicate the overall odds ratio of studies included.

Five RCTs reported the FBG levels of 414 patients, i.e., 207 glucomannan users and 207 nonusers [20,21,22,23,25]. The FBG levels between the two groups was significantly different (MD −1.08 [95% CI: −1.65, −0.50], *p* = 0.0002). The heterogeneity among the included RCTs was significantly different (*p* = 0.004, I^2^ = 74%) (Figure 5A).

2. Two-hour Postprandial Blood Glucose (P2hBG)

Four RCTs reported the P2hBG levels of 392 patients, i.e., 196 glucomannan users and 196 nonusers [21,22,23,25]. The P2hBG levels between the two groups were significantly different (MD −1.92 [95% CI: −3.19, −0.65], *p* = 0.003). The heterogeneity among the included RCTs was significantly different (*p* = 0.0005, I^2^ = 83%) (Figure 5B).

3. Fasting Insulin (FINS)

Three RCTs reported the FINS levels of 122 patients, i.e., 61 glucomannan users and 61 nonusers [20,21,25]. Glucomannan reduced the FINS levels, and the difference was significant (MD −1.59 [95% CI: −2.69, −0.50], *p* = 0.004). The heterogeneity among the included RCTs was not significantly different (*p* = 0.59, I^2^ = 0%) (Figure 5C).

4. Serum Fructosamine (SFRA)

Two RCTs reported the SFRA levels of 62 patients, i.e., 31 glucomannan users and 31 nonusers [20,25]. Glucomannan reduced the SFRA levels compared with controls, and the difference was significant (SMD −1.19 [95% CI: −1.74, −0.64], *p* < 0.0001). The heterogeneity among the included RCTs was not significantly different (*p* = 0.56, I^2^ = 0%) (Figure 5D).

#### 3.1.5. The Effect on the Physical Examination Index

1. Body Weight (BW)

**Figure 6:**
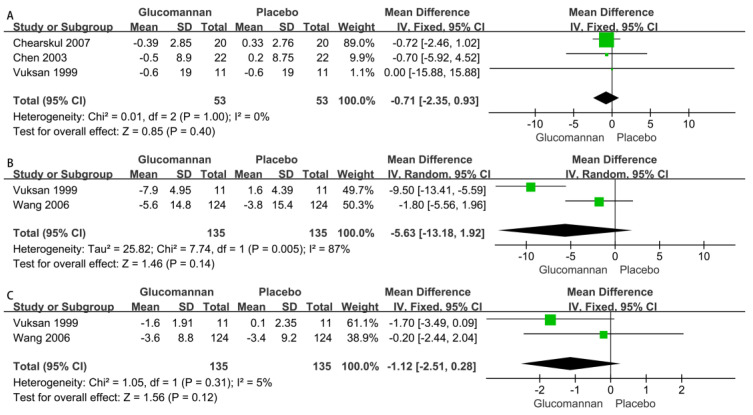
Forest plot of studies [20,22,23,25] on the effect of glucomannan intake on physical examination index included in this analysis. The effects of glucomannan on BW (**A**), SBP (**B**), and DBP (**C**), where green boxes indicate the odds ratio of the original research data and black diamonds indicate the overall odds ratio of studies included.

Three RCTs reported the BW of 106 patients, i.e., 53 glucomannan users and 53 nonusers [20,22,25]. The change in BW between the two groups was not significantly different (MD −0.71 [95% CI: −2.35, 0.93], *p* = 0.40). The heterogeneity among the studies was not significantly different (*p* = 1.00, I^2^ = 0%) (Figure 6A).

2. Systolic Blood Pressure (SBP)

Two RCTs reported the SBP of 270 patients, i.e., 135 glucomannan users and 135 nonusers [20,23]. The change in SBP between the two groups was not significantly different (MD −5.63 [95% CI: −13.18, 2.92], *p* = 0.14). The heterogeneity among the studies was significantly different (*p* = 0.005, I^2^ = 87%) (Figure 6B).

3. Diastolic Blood Pressure (DBP)

Two RCTs reported the DBP of 270 patients, i.e., 135 glucomannan users and 135 nonusers [20,23]. The change in DBP between the two groups was not significantly different (MD −1.12 [95% CI: −2.51, 0.28], *p* = 0.12). The heterogeneity among the studies was not significantly different (*p* = 0.31, I^2^ = 5%) (Figure 6C).

#### 3.1.6. Risk of Publication Bias

We used Egger’s test to determine the risk of publication bias. The results of the risk of publication bias are presented in the Supplementary Material (The results of the risk of publication bias). Only indicators containing three or more studies could be assessed for risk of publication bias, so SFRA, SBP, and DBP failed to be assessed. Among indicators being assessed, only with FBG did there exist a risk of publication bias (*p* = 0.030).

## 4. Discussion

In this meta-analysis, which included six RCTs, we fully evaluated the effects of glucomannan on patients with type II diabetes, with multidimensional indicators of diabetes, such as blood lipid levels, glycemic index, BP, and BW. Our analysis results showed that glucomannan had significant reduction effects on TC, LDL, FBG, P2hBG, FINS, and SFRA. Glucomannan might have reduction effects on HDL, BW, SBP, and DBP, but the differences were not statistically significant. Glucomannan had no reducing effect on TG.

As shown in Figure 4, glucomannan had a reducing effect on serum lipids. Glucomannan significantly reduced TC and LDL levels in patients with type II diabetes but had no effect on TG. The effects of glucomannan on blood lipid levels need to be confirmed in further studies.

As shown in Figure 5, glucomannan supplementation reduced glycemic indices, including FBG, P2hBG, FINS, and SFRA. Although differences were present in the background of the antidiabetic drugs, including in the levels of physical exercise and the dosage of glucomannan supplements, the consistency in lowering the blood glucose index was obvious. These results indicate that glucomannan supplementation has beneficial effects on the blood glucose index in type II diabetes. Serum HbA1c and serum fructosamine (SFRA) can only represent the blood glucose concentration from 6–8 weeks and 1–3 weeks before the measurement time, respectively [26]. SFRA is closely related to both serum HbA1c and blood glucose and may be a useful biomarker in clinical and epidemiological studies to test blood glucose levels [26]. In our study, glucomannan had a significant reduction effect on serum SFRA, which was consistent with the effects on FBG, P2hBG, and FINS.

As shown in Figure 6, glucomannan supplementation reduced BW, SBP, and DBP, but the change in BW, SBP, and DBP between the two groups was not significantly different. Glucomannan can reduce the absorption of nutrients by forming a defensive covering on the surface of the intestine, which reduces BP and BW [27]. However, the results of one RCT [28] showed that glucomannan supplementation had no effect on BW reduction in children with overweight and obesity. Conversely, in patients with type II diabetes, three RCTs [1,29,30] decreased BW, but the difference was not significant. The weight-reducing effect of glucomannan was inconsistent in different populations. To accurately determine the weight-decreasing and antihypertensive effects in a specific population, especially type II diabetes patients, it is necessary to expand the sample size in future studies.

In our meta-analysis, we used the fixed effects model when I^2^ did not exceed 50%, which indicated that heterogeneity was low. On the contrary, when I^2^ exceeded 50% we used the random effects model. For indicators using the random effects model, we conducted sensitivity analysis to explore the reasons of heterogeneity. After removing each study in turn, merged results of the remaining studies did not have significant differences compared with the original results, indicating that the original results were not likely to change. 

In this meta-analysis, the six RCTs were mainly performed in Asian countries and Canada in North America. The main researchers were mostly Asians, which might be related to the fact that konjac is mainly produced in Asia. The geographical and climatic environment in some parts of Asia is suitable for its growth, and the consumption of konjac has a long history in Asia. The main effective component of konjac is glucomannan. Specific doses of glucomannan were used in the six RCTs, making them more accurately studied than in usual food interventional research. The six RCTs were published between 1999 and 2007. No standard RCT results have been published in the past decade, but this might not hinder the authenticity and reliability of the results of this study. Glucomannan was a research hotspot for some time, and it may become a research hotspot again in the future.

The research population mainly comprised adults with type II diabetes and included slightly more women than men, totaling 440 people. The daily doses of the glucomannan supplement in the experimental group ranged from 1.2 g to 15 g, and the duration of the experiment ranged from 3 to 16 weeks. The placebo or wheat bran was used in the control group. As the main component of konjac, glucomannan did not cause a definite adverse reaction during the study. However, to determine the safety and effectiveness of a drug ingredient, studies need a longer experimental time.

Asian countries, represented by China, have a long history of consuming konjac food. Its effect of reducing weight was known before, but it had not been verified by scientific experiments before modern science confirmed its effective ingredients. Amorphophallus konjac is a natural food in which glucomannan, its main effective ingredient, can be naturally derived. The RCTs with the highest levels of evidence can directly and accurately verify glucomannan’s clinical effects. The effects of glucomannan on obesity, metabolic syndrome, and constipation have been studied. We carried out our study on the effects of glucomannan supplementation on type II diabetes in this research.

The treatments for type II diabetes mainly include diet control, exercise regimens, oral medication, insulin therapy, and the recent trend of surgery. Considering the poor compliance with diet and exercise therapy, the side effects of long-term oral medication and insulin therapy, and severe trauma after surgical treatment, glucomannan exhibits certain advantages as a natural product for nutritional intervention.

Glucomannan solution can also be dehydrated and properly treated to form edible and naturally degradable membrane materials [31]. As a natural polysaccharide with a unique structure and physical and chemical properties, glucomannan has a high application value and prospects in the areas of food, cosmetics, and biomedicine. Glucomannan has potential medical functions in weight loss, lipid reduction, sugar control, and intestinal probiotics. The excellent water absorption and satiety of glucomannan make it exhibit the characteristics of dietary fiber, which can stimulate intestinal transport and reduce constipation [32,33]. The intake of a low-fiber diet containing glucomannan in healthy adults can increase the frequency of defecation, reduce rectal pressure, and prevent gastrointestinal diseases [34]. Prebiotics are a kind of selective fermentation ingredient, which can promote the proliferation of beneficial microorganisms in the human gastrointestinal tract and benefit the health of the host. Beneficial bacteria can play an active role in human physiology, metabolism, nutrition, and immunity, and prevent the invasion and proliferation of pathogens. Glucomannan and its hydrolysate oligosaccharides can selectively regulate the number and distribution of beneficial intestinal microflora (lactic acid bacteria, bifidobacteria, cecal anaerobes, etc.), reduce the incidence of colon cancer, and have excellent prebiotic effects [34,35,36,37]. In the near future, related products with glucomannan as the main raw material, such as some medical functional foods, will gradually enter the market and become known to the public. This meta-analysis of the effects of glucomannan on patients with type II diabetes is based on this research and development trend of glucomannan.

Based on our literature search for this meta-analysis, we comprehensively evaluated for the first time the effects of glucomannan supplementation on various indicators in patients with type II diabetes. However, we failed to avoid several limitations: (a) Due to the limited number of large clinical trials, we included only six eligible RCTs, and there were only two studies including data of SFRA, SBP, and DBP. Most RCTs had a small sample size, so the results may not have been significant. (b) The effectiveness and safety of the long-term use of glucomannan and dose of glucomannan were not verified, nor was the daily dose of glucomannan. (c) The RCTs were conducted only in Asia and Canada, so our study lacked research from Europe and Africa. These limitations need to be overcome in future studies. Even with the above limitations, our findings clearly lend support to the suggestion that the supplementation of glucomannan has an ameliorative effect on type II diabetes mellitus.

## 5. Conclusions

Our results indicate that glucomannan supplementation has significant reducing effects on the TC, LDL, FBG, P2hBG, FINS, and SFRA of type II diabetic patients. However, the beneficial effects of glucomannan on TG, HDL, BW, SBP, and DBP were not statistically significant. More RCTs with larger sample sizes, combined drugs, and longer experimental times are needed to further determine the effects of glucomannan on various indicators of type II diabetes patients. Perhaps glucomannan can then be developed into a highly effective natural product, thus benefiting more type II diabetes patients.

## Figures and Tables

**Table 1:** Characteristics of the Included Studies.

First Author, Year (Reference)	Location	Cases	Controls	Age	Gender, Males	Dose	Duration	Comparator	Energy Balance
Vuksan, 1999 [20]	Canada	11	11	60.4 ± 7.53	45.5%	0.7 g/100 kcal	16 weeks	Wheat bran	Isocaloric
Liu, 2002 [21]	China	30	30	52.1 ± 4.89	43.3%	6 g/d	1 month	Placebo	Isocaloric
Chen, 2003 [22]	Taiwan	22	22	64.2 ± 8.3	45.5%	1.2–3.6 g/d	4 weeks	Placebo	Isocaloric
Wang, 2006 [23]	China	124	124	57.7 ± 9.16	43.0%	15 g/d	12 weeks	Placebo	Isocaloric
Yoshida, 2006 [24]	Canada	13	13	56.8 ± 10.8	30.8%	10 g/d	3 weeks	Placebo	Isocaloric
Chearskul, 2007 [25]	Thailand	20	20	51.2 ± 2.21	50.0%	3 g/d	4 weeks	Placebo	Isocaloric

## Data Availability

The data used to support the findings of this study are included in the article.

## Supplementary Materials

The following suporting information can be downloaded at: https://www.mdpi.com/article/10.3390/nu15030601/s1: The results of the risk of publication bias.
