# Supplementary material for: Effects of Glucomannan Supplementation on Type II Diabetes Mellitus in Humans: A Meta-Analysis
Source: Nutrients. 2023 Jan 24;15(3):601. doi: 10.3390/nu15030601 (PMC9919128; doi:10.3390/nu15030601)
Supplement: Supplementary file 1 [file nutrients-15-00601-s001.zip › nutrients-2154993-supplementary.pdf]

Supplementary information for

Effects of Glucomannan Supplementation on Type II Diabetes Mellitus in Humans: a Meta-Analysis

Zhanzhi Zhang[1,2,†], Yu Zhang[3,†], Xiaomei Tao[4,†], Yuying Wang[1,2], Benqiang Rao[1,2*], and Hanping Shi[1,2*]

1 Department of General Surgery, Beijing Shijitan Hospital, Capital Medical University, Beijing, 100038, China

2 Key Laboratory of Cancer FSMP for State Market Regulation, Beijing, 100038, China

3 Department of VIP Medical Services, National Cancer Center/National clinical Research Center for Can-cer/Cancer Hospital, Chinese Academy of Medical Sciences and Peking Union Medical College, Beijing 100021, China

4 Department of Pharmacy, Beijing Shijitan Hospital, Capital Medical University, Beijing, 100038, China

* Correspondence: raobenqiang@bjsjth.cn (B.R.); shihp@ccmu.edu.cn (H.S.)

† These authors contributed equally to this work.

Egger's test results for:

1. TG

```
. metabias WMD seWMD, egger

Note: data input format theta se_theta assumed

Egger's test for small-study effects:
Regress standard normal deviate of intervention
  effect estimate against its standard error

.
Number of studies =   6                                   Root MSE      =    2.123
------------------------------------------------------------------------------
     Std_Eff | Coefficient  Std. err.      t    P>|t|     [95% conf. interval]
-------------+----------------------------------------------------------------
       slope |   .0668407   .3097372     0.22   0.840    -.7931276    .9268091
        bias |  -.2884644   2.139682    -0.13   0.899    -6.229173    5.652244
------------------------------------------------------------------------------

Test of H0: no small-study effects          P = 0.899
```

2. TC

```
. metabias WMD seWMD, egger

Note: data input format theta se_theta assumed

Egger's test for small-study effects:
Regress standard normal deviate of intervention
  effect estimate against its standard error

.
Number of studies =   6                                   Root MSE      =    2.124
------------------------------------------------------------------------------
     Std_Eff | Coefficient  Std. err.      t    P>|t|     [95% conf. interval]
-------------+----------------------------------------------------------------
       slope |  -.1069534   .2111928    -0.51   0.639    -.6933186    .4794118
        bias |  -1.949235   1.851461    -1.05   0.352    -7.089714    3.191244
------------------------------------------------------------------------------

Test of H0: no small-study effects          P = 0.352
```

3. HDL

```
. metabias WMD seWMD, egger

Note: data input format theta se_theta assumed

Egger's test for small-study effects:
Regress standard normal deviate of intervention
  effect estimate against its standard error

.
Number of studies =   6                                   Root MSE      =    1.045
------------------------------------------------------------------------------
     Std_Eff | Coefficient  Std. err.      t    P>|t|     [95% conf. interval]
-------------+----------------------------------------------------------------
       slope |  -.0198727   .0323349    -0.61   0.572    -.1096486    .0699033
        bias |   .0358645   1.005967     0.04   0.973    -2.757148    2.828877
------------------------------------------------------------------------------

Test of H0: no small-study effects          P = 0.973
```

4. LDL

```
. metabias WMD seWMD, egger

Note: data input format theta se_theta assumed

Egger's test for small-study effects:
Regress standard normal deviate of intervention
  effect estimate against its standard error

.
Number of studies =  5                              Root MSE      =   1.693
------------------------------------------------------------------------------
     Std_Eff | Coefficient  Std. err.      t    P>|t|     [95% conf. interval]
-------------+----------------------------------------------------------------
       slope |  -.0326876   .1759782    -0.19   0.864    -.5927289    .5273536
        bias |   -3.40185   2.086358    -1.63   0.201    -10.04157    3.237871
------------------------------------------------------------------------------

Test of H0: no small-study effects          P = 0.201
```

5. FBG

```
. metabias WMD seWMD, egger

Note: data input format theta se_theta assumed

Egger's test for small-study effects:
Regress standard normal deviate of intervention
  effect estimate against its standard error

.
Number of studies =  5                              Root MSE      =   .9195
------------------------------------------------------------------------------
     Std_Eff | Coefficient  Std. err.      t    P>|t|     [95% conf. interval]
-------------+----------------------------------------------------------------
       slope |    .336673   .3101145     1.09   0.357    -.6502498    1.323596
        bias |  -4.134289   1.056561    -3.91   0.030    -7.496736   -.7718422
------------------------------------------------------------------------------

Test of H0: no small-study effects          P = 0.030
```

6. P2hBG

7. FINS

```
. metabias WMD seWMD, egger

Note: data input format theta se_theta assumed

Egger's test for small-study effects:
Regress standard normal deviate of intervention
  effect estimate against its standard error

.
Number of studies =  4                               Root MSE      =    1.74
------------------------------------------------------------------------------
     Std_Eff | Coefficient  Std. err.      t    P>|t|     [95% conf. interval]
-------------+----------------------------------------------------------------
       slope |   .3784644   .9440902     0.40   0.727    -3.683628    4.440557
        bias |  -4.149487   2.099595    -1.98   0.187    -13.18332    4.884343
------------------------------------------------------------------------------

Test of H0: no small-study effects          P = 0.187
```

8. BW

```
. metabias WMD seWMD, egger

Note: data input format theta se_theta assumed

Egger's test for small-study effects:
Regress standard normal deviate of intervention
  effect estimate against its standard error

.
Number of studies =  3                               Root MSE      =   .0463
------------------------------------------------------------------------------
     Std_Eff | Coefficient  Std. err.      t    P>|t|     [95% conf. interval]
-------------+----------------------------------------------------------------
       slope |  -.7905337   .0627152   -12.61   0.050    -1.587405    .0063378
        bias |   .0703693   .0432554     1.63   0.351    -.4792424     .619981
------------------------------------------------------------------------------

Test of H0: no small-study effects          P = 0.351
```
